# RNA Sequence Profiling Reveals Unique Immune and Metabolic Features of Breast Cancer Brain Metastases

**DOI:** 10.3389/fonc.2021.679262

**Published:** 2021-08-26

**Authors:** Limei Xiao, Jie Zhou, Hongyi Liu, Yuanyuan Zhou, Weibin Chen, Wugeng Cui, Yilin Zhao

**Affiliations:** ^1^School of Medicine, Xiamen University, Xiamen, China; ^2^Department of Oncology, Xiang’an Hospital of Xiamen University, School of Medicine, Xiamen University, Xiamen, China; ^3^School of Medical Science, Ningbo University, Ningbo, China; ^4^Department of Oncology and Vascular Interventional Radiology, Zhongshan Hospital, Xiamen University, Xiamen, China; ^5^Fujian Provincial Key Laboratory of Chronic Liver Disease and Hepatocellular Carcinoma (Xiamen University Affiliated ZhongShan Hospital), Xiamen, China

**Keywords:** BCBMs, BCs, OXPHOS, immunosuppression, extracranial metastases

## Abstract

There is an urgent need to improve our understanding of breast cancer brain metastases (BCBMs). Thus, we obtained transcriptome data of BCBMs, primary breast cancers (BCs), and extracranial metastases (BCEMs) from the Gene Expression Omnibus (GEO) database, including GSE43837, GSE14017, and GSE14018, for immune and metabolic analysis. Firstly, we performed immune and metabolic analysis on BCBMs and primary breast cancers of GSE43837 using RNA sequence. We identified significant immunosuppression and gene signatures associated with immune infiltration in BCBMs; the lower the expression of the signatures, the worse the prognosis of breast cancer patients in the Kaplan–Meier (KM) plotter [Breast cancer] database. We also identified increased oxidative phosphorylation (OXPHOS) utilization in BCBMs compared with BCs and gene signatures associated with increased OXPHOS utilization in BCBMs; the higher the expression of the signatures, the worse the prognosis of breast cancer patients in the KM plotter [Breast cancer] database, which can predict the prognosis of breast cancer patients better, as it can also predict the prognosis of patients with different breast cancer subtypes. In addition, we performed immune and metabolic analysis on BCBMs and extracranial metastases of GSE14017 and GSE14018 using RNA sequence. Compared with extracranial metastases, we identified more significant immunosuppression but no difference in OXPHOS utilization in BCBMs, which may be because OXPHOS was also involved in extracranial metastases. We have proven that OXPHOS was functionally significant in metastasis *in vitro* assays. Oligomycin, an OXPHOS inhibitor, substantially attenuated the migration and invasion potential of breast cancer cells. Our study provides new insights into the pathogenesis of BCBMs.

**Significance:**

Our study reports the most comprehensive gene expression analysis of BCBMs, BCs and extracranial metastases to date. We identified immunosuppression and OXPHOS enrichment in BCBMs compared with BCs, which provide new insights into the pathogenesis of BCBMs and will facilitate the development of new therapeutic strategies for patients with BCBMs.

## Introduction

Breast cancer is one of the most common causes of brain metastases (1, 2). Brain metastases usually occur in advanced breast cancer, and its prognosis is poor. The median overall survival time after development of brain metastases in breast cancer patients is approximately 7.4 months (range: 3.9–17.1 months) (3). Thus, it is an unmet clinical need to identify the underlying pathogenesis of BCBMs to develop rational therapeutic strategies.

In the past, the brain was considered an organ with immune privilege. However, many studies have shown that this immune privilege is not absolute, but relative to the immune privilege of other organs (4). The destruction of blood–brain barrier (BBB) by central nervous system tumors and the changes of extracellular matrix composition can make BBB leak at the tumor site (5). The intact brain contains almost no lymphocytes; However, T and B cells have been observed in the environment of brain metastasis (6). PD-1 inhibitors also showed activity against brain metastasis in patients with melanoma and lung cancer (7). Therefore, we must consider the unique characteristics of BCBMs compared with primary tumors and extracranial lesions prior to treatment with immunomodulatory therapy.

There is growing evidence that BCBMs possess different molecular characteristics compared with primary tumors and extracranial metastases. Other investigators’ whole exome sequencing study has detected the mutational signatures indicative of HRD scores increased in BCBMs compared with patient-matched primary tumors (8). Previous genomic analysis also identified mutations associated with sensitivity to PI3K/AKT/mTOR, CDK, and HER2/EGFR inhibitors in BCBMs compared with regional lymph nodes and extracranial metastases (9). Gene expression analysis identified that signatures indicative of BRCA1 deficiency were enriched in BCBMs compared with unmatched BCs (10).

However, there is no comprehensive immune and metabolic analysis on BCBMs, primary tumors, and extracranial metastases. This may be the reason why no significantly enriched pathways have been identified. In general, the mechanism of BCBMs is still unclear and needs to be further explored.

To address this urgent need, we collected gene expression profiles of BCBMs, BCs, and extracranial metastases from the GEO database: GSE43837 contained 19 BCBMs and 19 patient-unmatched BCs, GSE14017 contained 15 BCBMs and 14 extracranial metastases, and GSE14018 contained 7 BCBMs and 29 extracranial metastases. Together with functional assays on human breast cancer cell line (MDA-MB-231 cells), our study identified unique immune and metabolic features of BCBMs, which may contribute to develop new rational therapeutic strategies.

## Materials and Methods

### Procurement of RNA Sequencing Data and Batch Design

**Figure 1:**
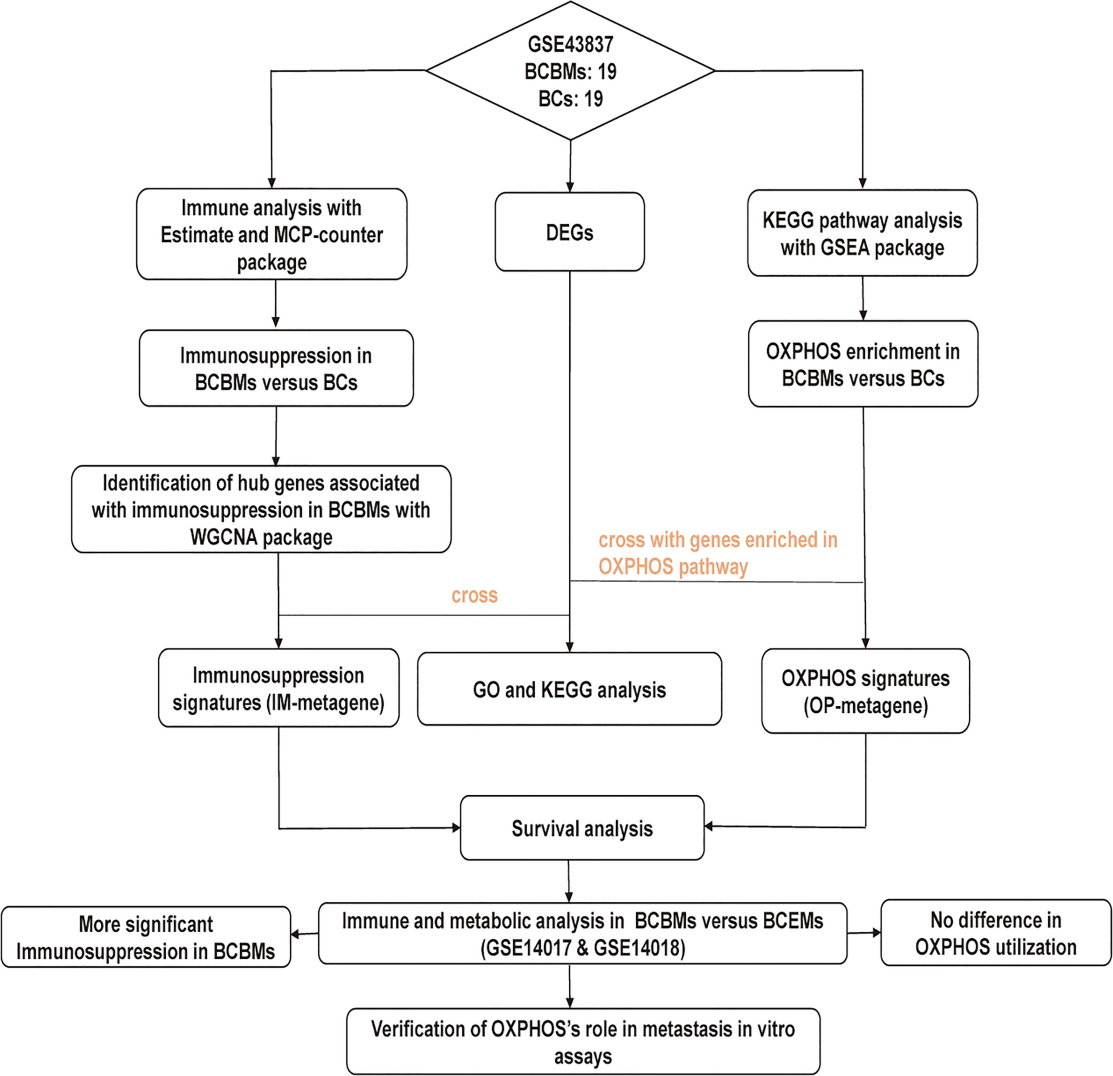
The workflow of the study. BCBMs, breast cancer brain metastases; BCs, breast cancers; DEGs, differentially expressed genes; OXPHOS: oxidative phosphorylation; IM-metagene, hub genes related to immune infiltration of BCBMs; OP-metagene, hub genes related to oxidative phosphorylation enrichment of BCBMs; GO, gene ontology; KEGG, kyoto encyclopedia of genes and genomes; BCEMs, breast cancer extracranial metastases.

The research strategy is presented in **Figure 1**. RNA sequencing data were downloaded from the National Central of Biology Information Gene Expression Omnibus (GEO) database (https://www.ncbi.nlm.nih.gov/geo/), including GSE43837, GSE14017, and GSE14018 (11). GSE43837 contains RNA sequence for 19 BCBMs and 19 BCs, GSE14017 contains RNA sequence for 15 BCBMs and 14 extracranial metastases (BCEMs), and GSE14018 contains RNA sequence for 7 BCBMs and 29 BCEMs. Microarray annotation information was used to match probes with corresponding genes. The median expression value was calculated out for the gene matched with more than one probe. We first performed the immune and metabolic analysis on BCBMs and BCs of GSE43837 and then performed a similar analysis on BCBMs and BCEMs of GSE14017 and GSE14018, respectively.

### Characterization of Immune Infiltration in BCBMs, BCs, and BCEMs

We utilized the Estimation of Stromal and Immune cells in Malignant Tumor tissues using Expression data (ESTIMATE) and Microenvironment Cell Populations-Counter (MCP-counter) R package to characterize immune infiltration in samples. ESTIMATE can infer the proportion of immune cells and stromal cells in tumor samples using gene expression (12). However, ESTIMATE cannot identify the distinct immune cell populations in heterogeneous tissues. In contrast, MCP-counter can quantify the absolute abundance of eight immune cells in heterogeneous tissues using transcriptome data (13).

### Construction of Co-Expression Network Associated With Immune Infiltration

WGCNA R package were used to construct a weight co-expression network associated with immune infiltration (14). First, based on the Pearson’s correlation value between paired genes, the expression levels of individual transcripts were converted into a similarity matrix. Next, we picked a proper soft threshold power that can increase strong correlations and decrease weak correlations between genes. The adjacency matrix was then converted into a topological overlap matrix when the soft threshold power *β* = 6. Then, the gene set was divided into several modules with similar expression patterns. Module–trait associations referred to the correlation between the module eigengene and the immune infiltration.

### Differentially Expressed Genes

Differentially expressed genes (DEGs) in different groups were identified using edgeR package (15). Specifically, edgeR adjusts gene expression according to different sequencing depths as represented by varying libraries. The Log2 fold-change (Log2FC) is an estimate of the log2 ratio of expression in a cluster to other clusters. A value of 1.0 indicates twofold greater expression in the cluster of interest. The exact test that adapted for the negative binomially distributed counts was chosen to judge the significance for DEGs. Adjusted *p*-values or false discovery rate (FDR) was determined by the default Benjamini–Hochberg (BH) correction in edgeR. For selecting the top features in a dataset, FDR < 0.05 and fold change (FC) > 1.5 were set as the cutoff criteria.

### Functional Enrichment Analysis of DEGs

The Database for Annotation, Visualization and Integrated Discovery (DAVID) v6.8 (https://david.ncifcrf.gov/summary.jsp) database integrates biological data and functional annotation tools to provide systematic and comprehensive biological function annotations for large-scale gene or protein lists. It was used to identify enriched Gene Ontology (GO) terms and Kyoto Encyclopedia of Genes and Genomes (KEGG) pathways of DEGs (16). *p*-values are determined by the Fisher’s exact test in DAVID. Adjusted *p*-values were determined by BH correction in DAVID. For selecting significant pathways, *p*-value < 0.05 and FDR < 0.25 were set as the cutoff criteria.

### Identification of Gene Signatures Associated With Immune Infiltration

We crossed the genes co-expressed with immune infiltration determined by the WGCNA package with DEGs to obtain gene signatures related to immune infiltration in the cluster of interest. The gene expression values for those signatures were then averaged to form the Immune metagene (IM-metagene). Specific genes were indicated in **Supplementary Table S4**.

### Gene Set Enrichment Analysis (GSEA)

GSEA determines whether an *a priori* defined set of genes has statistically significant difference in expression under two different biological conditions (17). GSEA software 3.0 downloaded from the Broad Institute was used for enrichment analysis for our datasets. The gene set of “c2.cp.kegg.v7.1.symbols.gmt”, which summarizes and represents specific, well-defined KEGG metabolic pathways, was downloaded from the Molecular Signatures Database (http://software.broadinstitude.org/gsea/msigdb/index.jsp). The normalized enrichment score (NES) represented the degree of enriched KEGG pathways in cluster of interest. *p*-values corresponding to each NES were determined by the Fisher’s exact test (1,000 permutations) in GSEA. Adjusted *p*-values were determined by BH correction in GSEA. For selecting significant pathways, FDR < 0.25 was set as the cutoff criteria.

### Identification of Gene Signatures Associated With OXPHOS Enrichment

We crossed core genes in OXPHOS enrichment in interested cluster determined by GSEA with DEGs to obtain signatures related to OXPHOS enrichment in the cluster of interest. The gene expression values for those signatures were then averaged to form the OXPHOS metagene (OP-metagene). Specific genes were indicated in **Supplementary Table S7**.

### Kaplan–Meier Plotter [Breast Cancer]

Kaplan–Meier (KM) plotter [Breast cancer] is an online survival analysis tool that can assess the prognostic function of 22,277 genes in breast cancer patients using microarray data (http://kmplot.com/analysis/index.php?p=background) (18). All KM plots were displayed using the “auto select best cutoff” parameter. Relapse-free survival (RFS), overall survival (OS), and distant metastasis-free survival (DMFS) were selected as the endpoints. Hazard ratio (HR) was considered significant when log rank *p*-value < 0.05. The corresponding 95% confidence intervals (95% CI) were also displayed on all KM plots.

### Cell Culture

MDA-MB-231 (human breast cancer cell line) cells were purchased from Procell Life Science & Technology Co. Ltd. MDA-MB-231 cells were cultured in Dulbecco’s modified Eagle’s medium (DMEM; BasalMedia, cat. no. L110KJ) supplemented with 10% fetal bovine serum (FBS; gibco, cat. no. A3160801) and 1% penicillin–streptomycin (BasalMedia, cat. no. S110JV) in a 95% humidified incubator containing 5% CO2 at 37°C.

### Cell Viability Assays

Cell proliferation assay. MDA-MB-231 cells (4 × 10^3^) were seeded on 96-well plates. After the cells adhered to the wall, the cells were treated with 1.0 µM oligomycin [Oligo(1.0)] and incubated in a 5% CO_2_ incubator at 37°C; 10 µl Cell Counting Kit-8 (CCK8; APEXBIO, cat. no. K1018) solution was then added into each well at 0 h, 12 h, 24 h, and 48 h, respectively, and cultured for 2 h. Next, the 96-well plates were put on the enzyme-linked immunoassay instrument and shaken for 2 s. The absorbance was measured at 460 nm. The growth rate was calculated as follows: Growth rate of Control = ABS(OD value of Control − mean(OD value of Control group))/mean(OD value of Control group), Growth rate of Oligo(1.0) = ABS(OD value of Oligo(1.0) − mean(OD value of Control group))/mean(OD value of Control group).

Cell apoptosis assay. Annexin V and propidium iodide (PI) (BD pharmingen, cat. no. 556547) were used to stain the cells cultured in medium. FSC-H and SSC-H of flow cytometry were used to detect single cells. The percentage of annexin V^−^/PI^−^ cells was used to represent the cell viability.

### Migration and Invasion Assays

Scratch assay. MDA-MB-231 cells (1 × 10^6^) were seeded on six-well plates. When the cell confluence reached 95%, the fused cells were scratched along the pore diameter with a sterile 200-µl pipette tip and then washed five times with PBS to remove floating cells and debris. The medium in each well was replaced with serum-free medium containing 1.0 µM oligomycin. The wound healing was observed at 0 and 48 h, and photos were taken under a microscope.

#### Transwell Assays

After starvation in serum-free medium for 6 h, cells were digested with 0.25% trypsin. The cell density was then adjusted to 2 × 10^5^/ml. One hundred microliters of cell suspension was added into the upper transwell chamber, and 180 µl of medium containing 10% FBS was added into the lower 24-well chamber to induce cell migration. Being allowed to migrate for 24 h, the cells on the lower surface of the upper chamber was immersed in 4% paraformaldehyde for 30 min, stained with crystal violet for 15 min, counted, and photographed under a microscope in the middle and four surrounding fields. For the invasion experiment, 3 × 10^4^ starved MDA-MB-231 cells were plated into the upper transwell chamber that was covered with 80 µl matrix glue (300 ng/ml). After 24 h, the invaded cells in the middle and four surrounding fields were counted and photographed under a microscope. The average number of cells in the five fields was used as the number of migrated and invaded cells.

## Results

### DEGs in BCBMs Compared With BCs

We used edgeR package to identify DEGs between BCBMs and BCs of GSE43837. A total of 539 DEGs were identified, of which 394 protein-coding genes were upregulated and 145 protein-coding genes were downregulated in BCBMs compared with BCs, respectively (FDR < 0.05, FC > 1.5; **Supplementary Table S1**).

### BCBMs and BCs Show Differences in Immune Cell Infiltration

**Figure 2:**
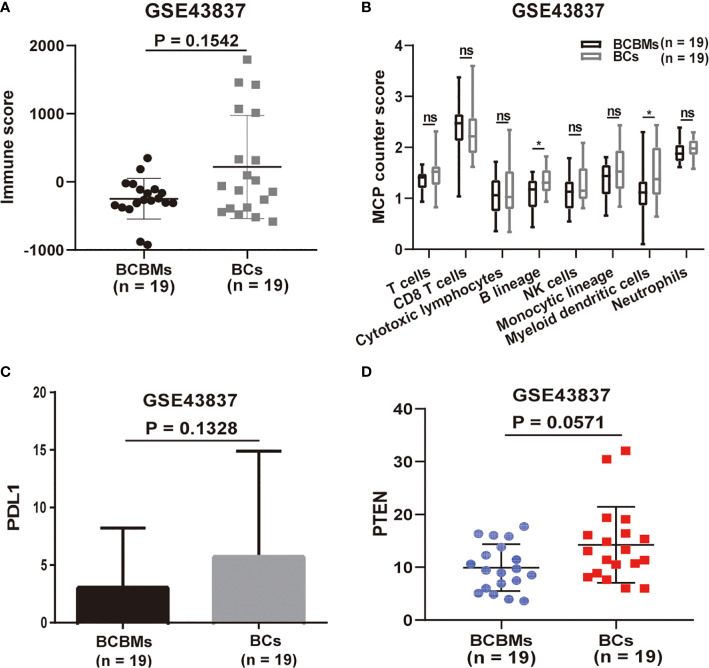
Immune infiltration heterogeneity in BCBMs compared with BCs (GSE43837). **(A)** ESTIMATE immune analysis of BCBMs (n=19) and BCs (n=19) (GSE43837). Lines represent mean ± SD, and each dot represents a single sample. Significance was determined via Wilcoxon rank-sum test. **(B)** MCP-counter analysis of indicated immune cell populations in BCBMs (n = 19) and BCs (n = 19) from GSE43837. Each plot is a simple box and whisker plot. Median values (lines) and interquartile range (whisker) are indicated. ns, not significant (P > 0.05); *P < 0.05. Significance was determined via a Wilcoxon rank-sum test. **(C)** Comparison of CPM for PDL1 RNA expression between BCBMs (n = 19) and BCs (n = 19) from GSE43837. Lines represent mean ± SD. Significance was determined via Wilcoxon rank-sum test. **(D)** Comparison of CPM for PTEN RNA expression between BCBMs (n = 19) and BCs (n = 19) from GSE43837. Lines represent mean ± SD, and each dot represents a single sample. Significance was determined via Wilcoxon rank-sum test.

Then, we performed immune analysis on BCBMs and BCs of GSE43837. We utilized the ESTIMATE and MCP-counter R packages to characterize differences in immune cell infiltration between BCBMs and BCs (GSE43837). ESTIMATE is a tool used to infer tumor purity and immune infiltration from gene-expression data that were originally validated in 11 cancer types (12). However, ESTIMATE can only assess the overall immune status of the tumor. On the contrary, MCP-counter can calculate the specific infiltration of T cells, CD8 T cells, cytotoxic lymphocytes, B lineage, NK cells, monocytic lineage, myeloid dendritic cells, and neutrophils in tumors based on gene expression (13). Together, ESTIMATE assessed that the immune score of BCBMs was lower than that of BC, although there was no statistical significance (*p* = 0.1542; **Figure 2A**); MCP-counter estimated that the infiltration of eight immune cells in BCBMs was also lower than that of BCs; in particular, the infiltration of B lineage (*p* < 0.05; **Figure 2B**) and myeloid dendritic cells (*p* < 0.05; **Figure 2B**) in BCBMs was significantly lower than that of BCs. As the immune infiltration is lower in BCBMs compared with BCs, and the expression of PDL1 and PTEN has been confirmed to be related to tumor immune infiltration in previous studies (19, 20), we also compared the expression of PDL1 and PTEN between BCBMs and BCs. The RNA expression of PDL1 was not different in BCBMs compared with BCs (*p* = 0.1328; **Figure 2C**). The RNA expression of PTEN in BCBMs was lower than that of BCs at the limit of significance (*p* = 0.0571; **Figure 2D**).

**Figure 3:**
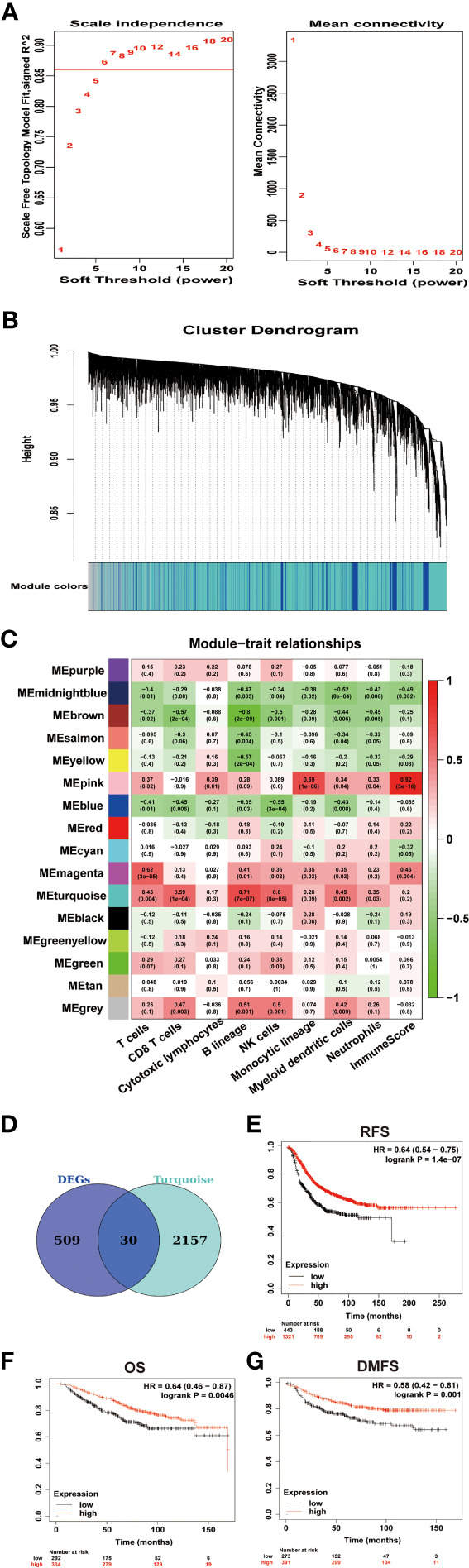
Identification of signatures associated with immune infiltration of BCBMs. **(A)** Analysis of network topology of GSE43837 dataset with different soft thresholds. The left panel shows the influence of soft threshold power (x-axis) on the scale-free fit index (y-axis). The right panel shows the influence of soft threshold power (x-axis) on mean connectivity (y-axis). **(B)** Dendrogram of gene clustering, the gene set was divided into 16 modules based on network topology. Different color modules contain different number of genes. **(C)** Heatmap shows correlations of module eigengenes with immune cell infiltration. Each cell contains the corresponding correlation and P value. **(D)** Venn diagram of DEGs and turquoise module eigengenes. A total of 30 overlapping genes were obtained. The full DEGs lists are provided in **Supplementary Table 1**. The 30 overlapping genes are provided in **Supplementary Table 4**. **(E–G)** Prognostic significances of IM-metagene in patients with breast cancer were shown based on the KM plotter database. RFS, relapse‐free survival; OS, overall survival; DMFS, distance metastasis free survival; and HR, hazard ratio. The P values were determined using a log-rank test.

**Figure 4:**
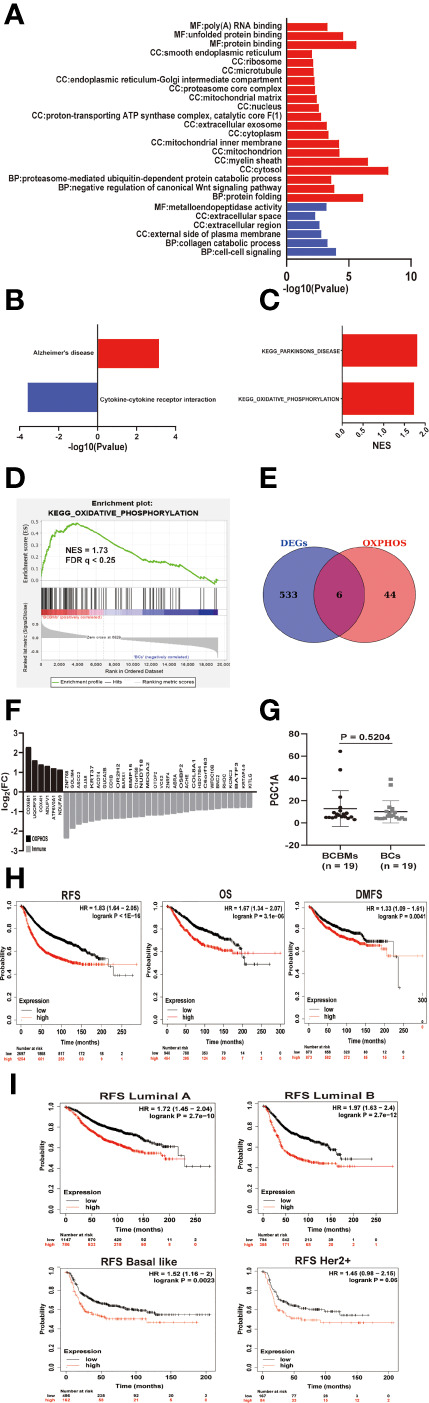
The metabolic features of BCBMs and survival analysis. **(A)** Gene ontology enrichment analysis of up-regulated (red) and down regulated (blue) genes in BCBMs (n = 19) versus BCs (n = 19) (GSE43837) (P < 0.05; FDR < 0.25). MF: Molecular function, CC: Cell component, BP: Biological process. **(B)** KEGG enrichment analysis of upregulated and down regulated gene sets in BCBMs (n = 19) versus BCs (n = 19) (GSE43837) (P < 0.05; FDR < 0.25). **(C)** GSEA analysis demonstrating all KEGG metabolism significantly altered (p < 0.05, FDR < 0.25) in BCBMs (n = 19) versus BCs (n = 19) (GSE43837). The normalized enrichment score (NES) forms the x-axis. Upregulated gene sets are shown in red. No down regulated gene sets met the criteria for statistical significance. **(D)** GSEA analysis enrichment plot demonstrating significant enrichment of OXPHOS gene set in BCBMs (n = 19) versus BCs (n = 19) (GSE43837). NES and FDR q are listed on the enrichment plot. **(E)** Venn diagram of DEGs and OXPHOS core enrichment genes obtained by GSEA. A total of 6 overlapping genes were obtained. The OXPHOS core enrichment genes obtained by GSEA are provided in **Supplementary Table 6**. The 6 overlapping genes are provided in **Supplementary Table 7**. **(F)** Bar graph showing log2(FC) values for differentially expressed OXPHOS- and immune-associated genes in BCBMs (n = 19) relative to BCs (n = 19). **(G)** Comparison of CPM for PGC1A RNA expression between BCBMs (n = 19) and BCs (n = 19) from GSE43837. Lines represent mean ± SD, and each dot represents a single sample. Significance was determined via Wilcoxon rank-sum test. **(H)** Prognostic significances of OP-metagene in patients with breast cancer were shown based on the KM plotter database. RFS, relapse‐free survival; OS, overall survival; DMFS, distance metastasis free survival; and HR, hazard ratio. The P values were determined using a log-rank test. **(I)** Prognostic significances of OP-metagene in patients with various breast cancer subtypes were shown based on the KM plotter database.

We used Weighted Correlation Network Analysis (WGCNA) R package to search for genes related to the immune infiltration of GSE43837. WGCNA R package is an effective tool that can be used to mine hub modules with similar expression patterns related to clinical traits (14). To build a scale-free network, we picked *β* = 6 (scale-free *R* ^2^ = 0.86) as the soft-thresholding power (**Figure 3A**). Then, those genes were classified into 16 modules (**Figure 3B**). As previous immune infiltration analysis identified B lineage and myeloid dendritic cell infiltration significantly decreased in BCBMs compared with BCs and the turquoise module had the highest correlation with B lineage (*r* = 0.71, *p* = 7e−07; **Figure 3C**) and myeloid dendritic cells (*r* = 0.49, *p* = 0.002; **Figure 3C**), the turquoise module was identified as a hub module significantly related to the immune infiltration of GSE43837 samples. To obtain the core immune signatures associated with BCBMs, we crossed the genes in turquoise module with DEGs. In total, we obtained 30 immune signatures (KRTAP4-9, BNC2, GUCA2B, BMP15, MDGA2, OTOP2, OSBP2, ZNF768, NUDT18, ABRA, KRT37, RHOC, COL8A1, GJA8, WFDC10B, GOLIM4, ASCC2, KITLG, ACOT4, BARX1, KCNC3, C6orf163, ACHE, HSD17B4, BATF3, CD1B, ZNRF4, C1orf158, OR2H2, and VCX2; **Figure 3D** and **Supplementary Table S4**), all of which were downregulated in BCBMs compared with BCs (**Figure 4F**). The gene expression values for all those signatures were then averaged to form the Immune metagene (IM-metagene).

Survival analysis for IM-metagene was performed in the KM plotter [Breast cancer] database. This was done to determine whether the expression of IM-metagene is related to the biological malignant behavior of breast cancer and whether IM-metagene can be used as a prognostic indicator for patients with breast cancer. KM plotter [Breast cancer] showed a significant decrease of RFS (HR = 0.7, log rank *p* = 5.3e−06), OS (HR = 0.66, log rank *p* = 0.011), and DMFS (HR =0.66, log rank *p* = 0.012) with lower expression of IM-metagene in patients with breast cancer (**Figures 3E–G**). According to molecular classification, breast cancer is divided into three subtypes: luminal epithelial type (luminal type), HER2 overexpression (HER2+) type, and basal-like type. Basal-like type molecules are expressed as ER(−)/PR(−)/HER2(−), which is equivalent to triple-negative breast cancer. Different breast cancer subtypes could vary for the prognosis and adjuvant treatments. Further exploring the relationship between IM-metagene and the prognosis of patients with breast cancer subtypes, we did not identify significant correlation between the expression of IM-metagene and the prognosis of patients with different breast cancer subtypes.

### Oxidative Phosphorylation Is Enriched in BCBMs Compared With BCs

To explore the biological and metabolic features of BCBMs, we used the DAVID tool to analyze the enrichment of GO and KEGG pathways of DEGs. DAVID is an online tool to analyze the biological function and the enrichment of KEGG pathways using gene lists. However, biological regulation is a progressive relationship; small changes in upstream genes may lead to obvious changes in downstream genes. If you use a set threshold to screen DEGs and then perform function/pathway enrichment analysis (GO/KEGG) directly, some gene information will be lost, which may result in missing significant biological and metabolic pathways. Therefore, we performed GSEA in the GSE43837 dataset. GSEA does not require a fixed threshold to filter genes. It is a method based on all-gene expression analysis and avoids the shortcomings of traditional enrichment analysis methods. Because there were not many DEGs in BCBMs compared with BCs, if the threshold was set to FDR < 0.05, a lot of GO terms will be missed. Therefore, we set the screening conditions as *p*-value < 0.05, FDR < 0.25. The top GO terms for BCBMs included protein folding (CCT3, LRPAP1, TRAP1, LMAN2L, NFYC, TBCC, DNAJB2, GNAO1, MLEC, ERP27, CCT7, CRYAB, PPIA, PFDN5, SIL1, and AARS; **Figure 4A** and **Supplementary Table S2**) and negative regulation of canonical Wnt signaling pathway (PSMB6, PSMB4, PSMB2, FRZB, HDAC1, DDIT3, PSMD2, UBC, PSMB1, KREMEN2, SOX9, and PFDN5; **Figure 4A** and **Supplementary Table S2**). Interestingly, GO analysis also showed that overexpressed genes in BCBMs compared with BCs were mainly enriched in cellular components related to oxidative phosphorylation (OXPHOS), such as mitochondria, mitochondrial matrix, mitochondrial inner membrane, proton transport ATP synthase complex, and catalytic core F (1) (*p* < 0.05, FDR < 0.25; **Figure 4A** and **Supplementary Table S2**); the top GO terms for BCs include cell–cell signaling (CCR1, CXCL10, GJB2, CXCL9, FGFBP1, CCL8, SH2D1A, IHH, and BARX1) and collagen catabolic process (MMP12, MMP11, COL3A1, MMP13, and MMP1) and other extracellular pathways (*p* < 0.05, FDR < 0.25; **Figure 4A** and **Supplementary Table S2**). KEGG pathway analysis using DAVID database showed that upregulated genes were enriched in Alzheimer’s disease and downregulated genes were enriched in cytokine–cytokine receptor interaction (*p* < 0.05, FDR < 0.25; **Figure 4B** and Supplementary 2) in BCBMs compared with BCs. GSEA detected the significant enrichment of Parkinson’s disease and OXPHOS (FDR < 0.25; **Figure 4C** and **Supplementary Table S5**) in BCBMs compared with BCs using the c2.cp.kegg.v7.1.symbols.gmt gene sets. Considering that the enrichment of Parkinson’s disease may be due to the contamination of the surrounding brain tissue, and the GO analysis identified many cell components related to OXPHOS enriched in BCBMs compared with BCs, our next step was mainly focused on OXPHOS (**Figure 4D**).

To obtain the core signatures related to OXPHOS enrichment of BCBMs, we crossed DEGs with 49 core genes in OXPHOS enrichment in BCBMs compared with BCs determined by GSEA (**Supplementary Table S6**), and obtained six signatures (COX6B1, UQCRFS1, COX4I1, NDUFV1, ATP6V0A1, and NDUFA9; **Figure 4E** and**Supplementary Table S7**), respectively. All the six OXPHOS signatures were upregulated in BCBMs compared with BCs (**Figure 4F**). The gene expression values for all those signatures were then averaged to form the OXPHOS metagene (OP-metagene).

Next, we performed a series of survival analyses in patients with breast cancer using microarray data in the KM Plotter [Breast cancer] database. KM plotter [Breast cancer] is an analytical database that can be used to determine whether gene expression is statistically related to the prognosis of breast cancer patients. This was done to determine whether OP-metagene was higher in more biologically aggressive tumors and whether it has value as predictive biomarkers for disease progression in patients. Remarkably, the analysis identified significant decrease of RFS (HR = 1.83, log rank *p* < 1e−16), OS (HR = 1.67, log rank *p* = 3.1e−06), and DMFS (HR = 1.33, log rank *p* = 0.0041) with higher expression of OP-metagene in breast cancers (**Figure 4H**). Then, we continued to explore whether OP-metagene is significantly associated with the prognosis of different breast cancer subtypes. The results showed that the higher the expression of OP-metagene, the shorter the RFS of breast cancer patients with luminal A (HR = 1.72, log rank *p* = 2.7e−10), luminal B (HR = 1.97, log rank *p* = 2.7e−12), basal-like (HR=1.52, log rank *p* = 0.0023), and HER2+ (HR = 1.45, log rank *p* = 0.06, in the edge of significance) (**Figure 4I**) subtypes, suggesting that OP-metagene may be a better biomarker for predicting disease progression in patients with breast cancer than IM-metagene. We also tried to explore the cause for the enrichment of OXPHOS in BCBMs compared with BCs. As a previous study has reported that PGC1A mediates mitochondrial biosynthesis and OXPHOS in cancer cells to promote metastasis (21), we compared the RNA expression of PGC1A in BCBMs and BCs, but there was no difference between the two clusters (*p* = 0.5204; **Figure 4G**).

### Immune and Metabolic Analysis in BCBMs and Extracranial Metastases

**Figure 5:**
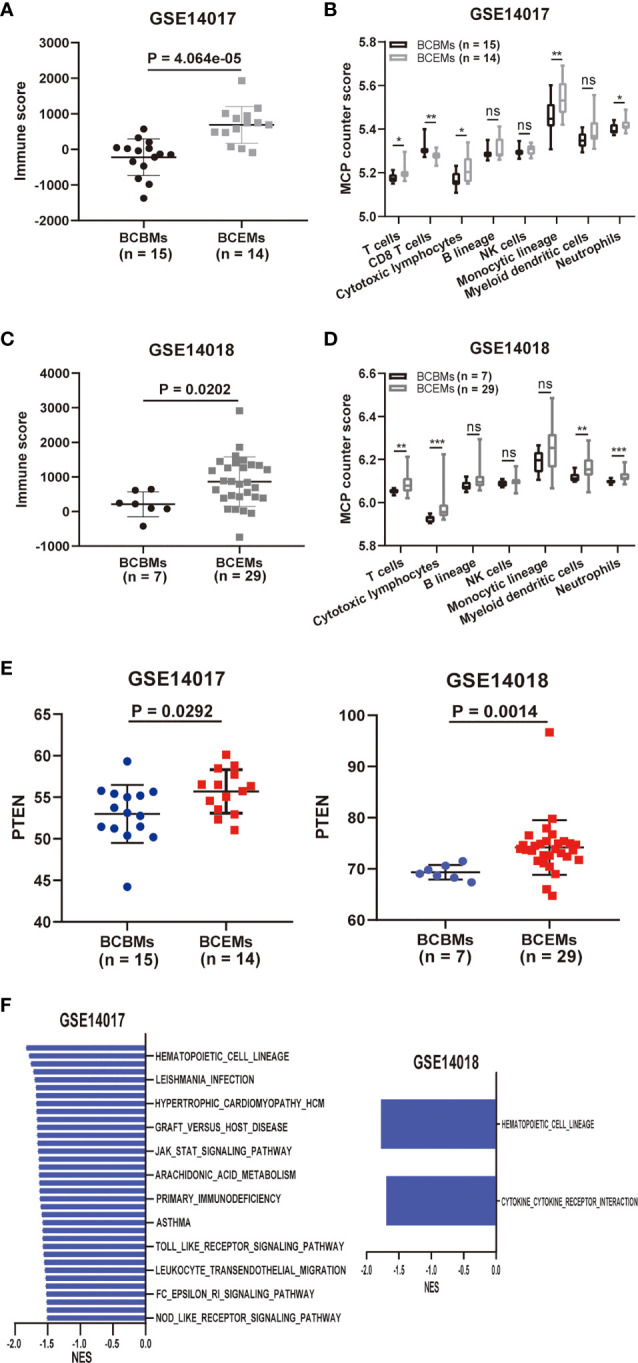
Comparison of immune and metabolic characteristics between BCBMs and BCEMs. **(A, B)** ESTIMATE immune and MCP-counter analysis of BCBMs (n = 15) and BCEMs (n = 14) (GSE14017). **(C, D)** ESTIMATE immune and MCP-counter analysis of BCBMs (n = 7) and BCEMs (n = 29) (GSE14018). **(E)** Comparison of CPM for PTEN RNA expression between BCBMs (n = 15) and BCEMs (n = 14) from GSE14017, BCBRs (n = 7) and BCEMs (n = 29) from GSE14018. Significance was determined via Wilcoxon rank-sum test. **(F)** GSEA analysis demonstrating all KEGG metabolism significantly altered (p < 0.05, FDR < 0.25) in BCBMs versus BCEMs (GSE14017 and GSE14018). The normalized enrichment score (NES) forms the x-axis. Downregulated gene sets are shown in red. No upregulated gene sets met the criteria for statistical significance. ns, not significant; *P < 0.05, **P < 0.01, ***P < 0.001, determined by Wilcoxon rank-sum test.

Exploratory immune and metabolic analysis was performed on BCBMs and extracranial metastases of GSE14017 and GSE14018, respectively, including lung metastases, bone metastases, and liver metastases. ESTIMATE-identified immune scores decreased significantly in BCBMs compared with BCEMs of GSE14017 (*p* = 4.064e−05; **Figure 5A**) and GSE14018 (*p* = 0.0202; **Figure 5C**) using RNA sequence. Specifically, MCP-counter-identified T cells (*p* < 0.05), Cytotoxic lymphocytes (*p* < 0.05), Monocytic lineage (*p* < 0.01), and Neutrophils (*p* < 0.05) infiltration decreased significantly in BCBMs compared with BCEMs of GSE14017 (**Figure 5B**), and there were more significant decreases in T cells (*p* < 0.01), Cytotoxic lymphocytes (*p* < 0.001), Myeloid dendritic cells (*p* < 0.01), and Neutrophils (*p* < 0.001) infiltration in BCBMs compared with BCEMs of GSE14018 (**Figure 5D**). However, MCP-counter failed to detect the infiltration of CD8 T cells of GSE14018. Exploratory PTEN RNA expression comparison was also performed on a small cohort RNA-seq of BCBMs versus BCEMs (GSE14017 and GSE14018). PTEN RNA expression was significantly decreased in BCBMs compared with BCEMs of GSE14017 (*p* = 0.0292; **Figure 5E**, left) and GSE14018 (*p* = 0.0014; **Figure 5E**, right). We also performed GSEA in BCBMs versus BCEMs. No upregulated pathway was discovered in BCBMs compared with BCEMs. Some immune-related pathways were discovered downregulated in BCBMs compared with BCEMs in GSE14017 and GSE14018 (FDR < 0.25, **Figure 5F** and **Supplementary Table S8**), such as antigen processing and presentation, leishmania infection, jak stat signaling pathway, and nod-like receptor signaling pathway.

### OXPHOS Is Functionally Significant for Metastasis

**Figure 6:**
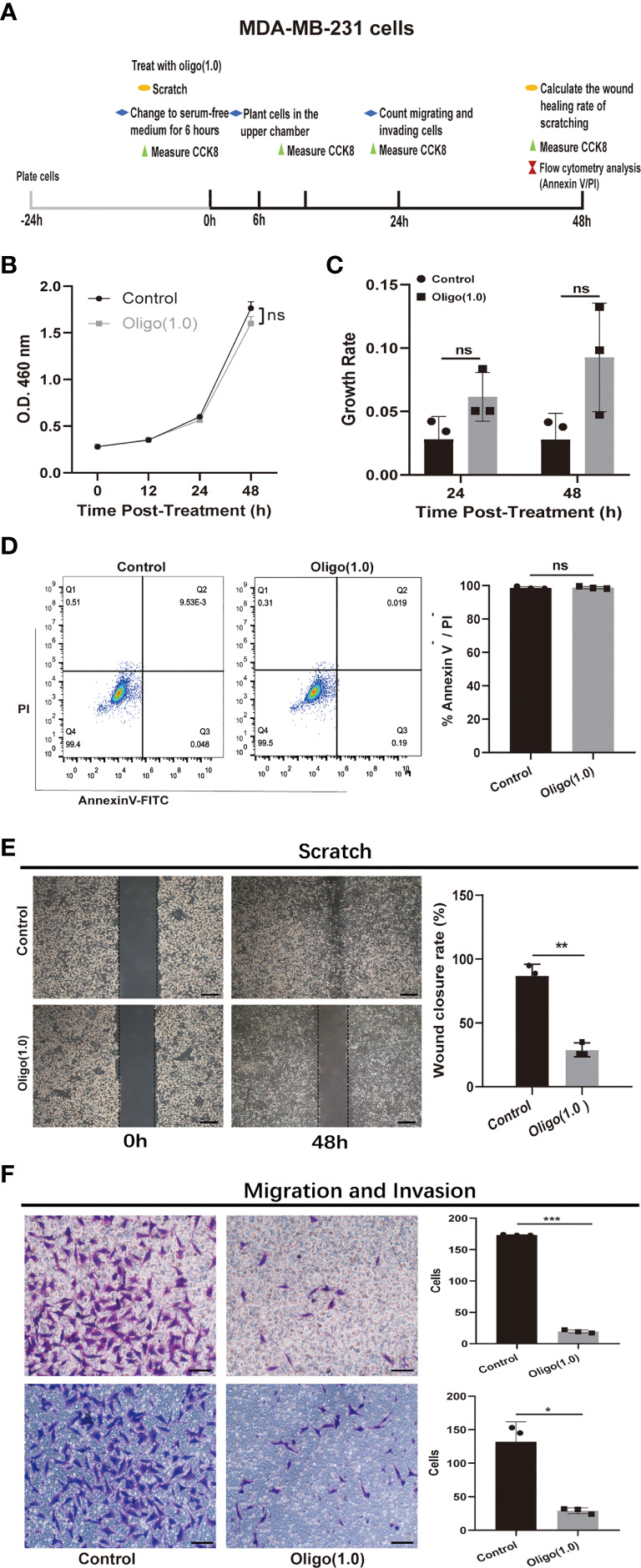
In vitro experiments confirmed that OXPHOS plays an important role in breast cancer metastases. **(A)** Schematic diagram of experimental plan for determining the effect of oligomycin treatment on MDA-MB-231 cells. Oligo(1.0) = 1 μM oligomycin. **(B, C)** The effect of Oligo(1.0) on the proliferation and growth rate of MDA-MB-231 cells measured by CCK8. The calculation method of growth rate was as follows: Growth rate of Control = ABS(OD value of Control – mean(OD value of Control group)) / mean(OD value of Control group), Growth rate of Oligo(1.0) = ABS(OD value of Oligo(1.0) - mean(OD value of Control group)) / mean(OD value of Control group). **(D)** Representative images of flow cytometry analysis of MDA-MB-231 cells treated with Oligo(1.0) after 48h. **(E)** Representative images of 48h wound healing rate of scratched wounds of MDA-MB-231 cells treated with Oligo(1.0). Scale bars = 200um. **(F)** Representative images of 24h migrating and invasion results of MDA-MB-231 cells treated with Oligo(1.0). Scale bars = 50 um. *P < 0.05, **P < 0.01, ***P < 0.001, ns not significant, P values determined by unpaired two-sided Student’s t-test. The data is expressed as the mean ± sd for n =3 replicates.

Next, we investigated whether increased OXPHOS utilization is functionally important for metastasis or only represents a response to the interplay between metastatic tumor cells and the brain microenvironment. We used oligomycin, an inhibitor of mitochondrial F(1)F(o)ATPase, to inhibit OXPHOS in MDA-MB-231 cells. Schematic diagram of experimental plan for determining the effect of oligomycin treatment on MDA-MB-231 cells was presented in **Figure 6A**. We first explored the effect of oligomycin on the proliferation and viability of MDA-MB-231cells using cell proliferation curve and flow apoptosis assays. The CCK8 proliferation curve showed that there was no significant difference in proliferation (*p* > 0.05; **Figure 6B**) and growth rate (*p* > 0.05; **Figure 6C**) of MDA-MB-231 cells in the control group and Oligo(1.0) group within 48 h. Flow cytometry analysis of cells stained with Annexin V and PI showed that compared with the control group, Oligo(1.0) did not reduce cell viability or increase cell apoptosis after 48 h (*p* > 0.05; **Figure 6D**). These results were consistent with previous studies that cancer cells can switch between glycolysis and OXPHOS to adapt to the environment (22). Then, we explored the effect of oligomycin on the metastatic potential of MDA-MB-231 cells using scratch, migration, and invasion assays. The scratch assay showed that the healing rate of scratch wounds significantly decreased in the Oligo(1.0) group compared with the control group (*p* < 0.01; **Figure 6E**). Migration and invasion assays showed that the migrated (*p* < 0.001; **Figure 6F**) and invaded cells (*p* < 0.05; **Figure 6F**) significantly decreased in the Oligo(1.0) group compared with the control group. Together, these assays confirmed that MDA-MB-231 cells can switch between glycolysis and OXPHOS to adapt to the environment and had stronger migration and invasion potential in the case of OXPHOS metabolism.

## Discussion

Although the treatments in BCs have been greatly improved, its outcome is not ideal. The drug resistance of BCs and the incidence of brain metastasis are gradually increasing (1). Therefore, it is critical to improve our understanding of the underlying immune and metabolic features that promote BCBMs, which can help for the development of more rational therapies for patients with BCs and/or BCBMs. To address this problem, we collected gene expression profiles of BCBMs, BCs, and extracranial metastases from the GEO database to perform immune and metabolic analysis.

We found significant immunosuppression in BCBMs compared with primary tumors using RNA sequence, a finding also observed by other investigators using IHC (23). We identified an IM-metagene associated with BCBM’s immunosuppression; its expression in BCBMs was significantly lower than that in BCs. In the KM plotter [breast cancer] database, the lower the expression of IM-metagene, the worse the prognosis of breast cancer patients. Moreover, we identified more significant immunosuppression in BCBMs compared with extracranial metastases using RNA sequence. This may be attributed to the immune escape mechanism of tumors and the differentiated immune environment of the brain. We also detected that PTEN RNA expression was significantly lower in BCBMs compared with primary tumors and extracranial metastases. Our results are consistent with previous studies that PTEN expression in tumors is inhibited by microRNA secreted by astrocytes in the brain, which is conducive to the growth of metastatic tumor and the formation of immunosuppression (19, 24). According to our results, mono immunotherapy may have limited effects in BCBMs, as previous studies have showed that sufficient infiltration of CD8 T cells and other immune cells is positively associated with the response of anti PD-L1 immunotherapy (20, 25, 26). Immunotherapy may need to be combined with chemotherapy and/or radiotherapy in patients with BCBMs, which can stimulate immune infiltration in a variety of ways. For example, low dose of cyclophosphamide can inhibit and deplete regulatory T cells and enhance the anti-tumor activities of CD4 T, CD8 T, natural killer (NK), or dendritic cells (27–29); 5-Fluorouracil and other p53-activating cytotoxic drugs can upregulate the expression and release of tumor-associated immunogen and enhance the antigen presentation function of dendritic cells (30, 31); antiangiogenic agents can improve the response of immunotherapy by targeting VEGF or VEGFR because VEGF can enhance expression of PD-1 and other inhibition checkpoints involved in CD8 T-cell exhaustion (32). Radiotherapy can induce damaged tumor cells to release numerous damaged DNA, tumor-associated antigens, and interferon type I, which can drive immune activation and inflammation (33). Some clinical trials have already proved the efficacy of immunotherapy combined with chemotherapy or radiotherapy. For example, local chemotherapy combined with systemic checkpoint blocking inhibitor (CTLA-4 blockade) has been shown to improve the prognosis of patients with melanoma (34). TG4010, a modified vaccinia Ankara, combined with chemotherapy seems to improve progression-free survival in non-small cell lung cancer (35). Moreover, pembrolizumab plus multisite stereotactic body radiotherapy has been proved to be well tolerated and to demonstrate clinical activity in patients with metastatic solid tumors (36). Therefore, it would be brilliant to develop rational combined immunotherapies in patients with BCBMs.

Our analysis also found that compared with nonmetastatic primary breast cancer, OXPHOS utilization was increased in BCBMs using RNA sequencing. We identified an OP-metagene that is enriched in BCBMs compared with BCs, and the KM plotter [Breast cancer] database confirmed that the high expression of OP-metagene was significantly correlated with poor RFS, OS, and DMFS of breast cancer patients (including different breast cancer subtypes). However, we did not detect significant difference in the RNA expression of PGC1A between BCBMs and BCs, which has been shown to mediate mitochondrial biosynthesis and OXPHOS in cancer cells to promote metastasis (21). That result is inconsistent with the traditional Warburg effect (aerobic glycolysis theory clouded); that is, tumor cells mainly depended on glycolysis to produce energy and promote cell growth, even in the presence of sufficient oxygen (37). However, we did not detect OXPHOS utilization difference in BCBMs compared with extracranial metastases, which may be because OXPHOS was also involved in breast cancer extracranial metastases. Many previous studies support this hypothesis. Other people confirmed that compared with the primary tumor, breast cancer lung metastases showed OXPHOS enrichment, and OXPHOS plays an important role in the cascade process of breast cancer cells from *in situ* to lung metastasis (38). It has also been reported that breast cancer cells enriched in OXPHOS were more prone to bone metastasis (39). Some drug studies have also shown that OXPHOS plays an important role in breast cancer metastases. For example, marizomib reduces the number of circulating tumor cells and the expression of epithelial–mesenchymal transition-related genes by inhibiting OXPHOS and proteasome in triple-negative breast cancer to reduce lung and brain metastases (40); CSC acquires hormone therapy (HT) resistance and mediates metastasis progression through activated OXPHOS metabolism in luminal breast cancer (41). Human epidemiology also supports the role of OXPHOS in cancer progression, suggesting that metformin (an inhibitor of mitochondrial complex I) can reduce the recurrence and metastasis of breast cancer (42). In support of this finding, we use a rigorous method to prove that MDA-MB-231 cells can switch between OXPHOS and glycolysis to adapt to the environment (22) and had stronger migration and invasion potential in the condition of OXPHOS metabolism.

OXPHOS can promote metastatic seeding in a variety of ways. OXPHOS may induce epithelial–mesenchymal transition progression of cancer cells (43, 44). The increase of ATP production by OXPHOS can provide energy for the movement of cytoskeleton and survive in the process of cell detachment and migration (45, 46). What is exciting is that there are already drugs targeting mitochondrial metabolism that can penetrate the BBB in clinical trials [e.g., IACS-010759 (47, 48)]. Moreover, studies have shown that metformin can affect the immune microenvironment of tumor and increase the activity and infiltration of CD8 + cytotoxic T lymphocytes and the production of immune cytokines (49, 50), which implied that the OXPHOS inhibitor can be combined with immune checkpoint inhibitors. Others have already reported that the combination of IACS-010759, XRT, and anti-PD-1 drugs can improve the efficacy of anti-PD-1 drugs and prolonged survival time of patients with anti-PD-1 tolerance (51).

In conclusion, our study identified immunosuppression in BCBMs compared with BCs and extracranial metastases using RNA sequence and an IM-metagene that can be used as a prognostic indicator of breast cancer patients in the KM plotter [Breast cancer] database. We also identified OXPHOS enrichment in BCBMs compared with nonmetastatic primary tumors using RNA sequence and an OP-metagene that can better predict the prognosis of patients with breast cancer than IM-metagene, as it can predict the prognosis of patients with various subtypes of breast cancer in the KM plotter [Breast cancer] database. However, we did not identify a significant difference in OXPHOS utilization in BCBMs compared with extracranial metastases, which may be because the increased utilization of OXPHOS not only is unique to BCBMs, but also plays an important role in extracranial metastases (38, 39, 45). We confirmed that strictly human breast cancer cell line MDA-MB-231 cells can switch between OXPHOS and glycolysis to adapt to the environment and had stronger migration and invasion potential in the condition of OXPHOS metabolism *in vitro* assays. Together, we identified immunosuppression and enrichment of OXPHOS in BCBMs compared with BCs, which provides ideas for the development of more reasonable treatment strategies for patients with BCBMs. Our results suggest that immunotherapy combined with chemotherapy, radiotherapy, and/or OXPHOS inhibitors may improve the prognosis of patients with BCBMs.

However, our study has some limitations. We did not verify our findings in animal experiments. We did not further link our findings to DNA alterations, which play a pivotal role in the clinical administration of BC patients, because we are unable to collect valid DNA sequence at present. We did not clarify whether immunosuppression simply represents a response to the brain microenvironment or is involved in the whole cascade process of brain metastases. We did not clarify the relationship between OXPHOS and immunosuppression in BCBMs. That will be the focus of our future efforts.

## Data Availability Statement

The authors declare that all data supporting the findings of this study are available within the article and its **Supplementary Information** files or from the corresponding author on reasonable request. All RNA-seq data files that were reanalysed here are available in the GEO database under the following accession codes: GSE43837, GSE14017 and GSE14018.

## Author Contributions

LX: Conceptualization, methodology, data curation, writing—original draft preparation, visualization, investigation, supervision, software, validation, and writing—reviewing and editing. JZ: Co-designing and performing the experiments. HL, YZ, and WC: Reagents and materials. YLZ and WC: Project administration and funding acquisition. All authors contributed to the article and approved the submitted version.

## Funding

Natural Science Foundation of China: 81770294.

## Conflict of Interest

The authors declare that the research was conducted in the absence of any commercial or financial relationships that could be construed as a potential conflict of interest.

## Publisher’s Note

All claims expressed in this article are solely those of the authors and do not necessarily represent those of their affiliated organizations, or those of the publisher, the editors and the reviewers. Any product that may be evaluated in this article, or claim that may be made by its manufacturer, is not guaranteed or endorsed by the publisher.

## Supplementary Material

Supplementary Table 1 539 differentially expressed genes (DEGs) between BCBMs and BCs. Differential expression analysis of BCBMs (n = 19) and BCs (n = 19) was performed using edgeR package. P values were determined by the exact test in edgeR. Adjusted P values were determined by the default Benjamini-Hochberg (BH) correction in edgeR.

Supplementary Table 2 GO and KEGG terms for the 539 DEGs between BCBMs and BCs. P values are determined by the Fisher’s exact test in DAVID. Adjusted P values were determined by Benjamini-Hochberg (BH) correction in DAVID.

Supplementary Table 3 Genes of the turquoise module that was significantly correlated with immune infiltration of the sample.

Supplementary Table 4 The results of DEGs crossed with genes in turquoise.

Supplementary Table 5 The results of gene set enrichment analysis in BCBMs compared with BCs (GSE43837). The normalized enrichment score (NES) represented the degree of enrichment of the KEGG pathway in BCBMs compared with BCs. P values corresponding to each NES were determined by the Fisher’s exact test (1000 permutation) in GSEA. Adjusted P values corresponding to each NES were determined by Benjamini-Hochberg (BH) correction in GSEA.

Supplementary Table 6 The core genes of oxidative phosphorylation pathway enriched in BCBMs compared with BCs.

Supplementary Table 7 The results of DEGs crossed with core genes in oxidative phosphorylation pathway enriched in BCBMs compared with BCs.

Supplementary Table 8 The results of gene set enrichment analysis in BCBMs compared with BCEMs (GSE14017 and GSE14018). The normalized enrichment score (NES) represented the degree of enrichment of the KEGG pathway in BCBMs compared with BCs. P values corresponding to each NES were determined by the Fisher’s exact test (1000 permutation) in GSEA. Adjusted P values corresponding to each NES were determined by Benjamini-Hochberg (BH) correction in GSEA.
